# The California Nutrition Incentive Program: Participants’ Perceptions and Associations with Produce Purchases, Consumption, and Food Security

**DOI:** 10.3390/nu14132699

**Published:** 2022-06-29

**Authors:** Wendi Gosliner, Sridharshi C. Hewawitharana, Ron Strochlic, Celeste Felix, Caroline Long

**Affiliations:** Nutrition Policy Institute, Division of Agriculture and Natural Resources, University of California, Oakland, CA 94607, USA; shewawitharana@ucanr.edu (S.C.H.); rstrochlic@ucanr.edu (R.S.); cmfelix@ucanr.edu (C.F.); celong@ucanr.edu (C.L.)

**Keywords:** supplemental nutrition assistance program, diet, nutrition, farmers’ market incentives, food security, food policy, poverty, fruits and vegetables

## Abstract

We examined the associations of a Supplemental Nutrition Assistance Program (SNAP) point-of-purchase financial incentive program at farmers’ markets with produce purchase, consumption, and food security outcomes. We conducted cross-sectional, interviewer-administered intercept surveys with 325 adult SNAP participants at six incentive programs, five comparison farmers’ markets, and nine comparison supermarkets in California in the summer of 2018. The program provided dollar-for-dollar point-of-purchase incentives with $10 or $20 maximum at participating farmers’ markets. We measured produce consumption by an NCI screener; food security by the USDA 6-item screener; and program satisfaction with open-ended questions asked of a subsample. The quantitative analysis involved multilevel linear and logistic regression, adjusted for covariates. Qualitative data were coded and analyzed thematically. Shoppers at farmers’ markets offering $20 incentives had significantly higher odds of purchasing most of their produce at farmers’ markets than shoppers at $10 incentive (3.1, CI: 1.1, 8.7) or comparison markets (8.1, CI 2.2, 29.7). Incentives were not associated with quantitatively measured produce consumption. Each additional incentive dollar was associated with reduced odds of food insecurity (0.987, CI 0.976, 0.999). Participants appreciated the program; supermarket shoppers lacked awareness. Point-of-purchase incentives are appreciated and underutilized. Further understanding of optimal program design for produce consumption and food security impact is needed.

## 1. Introduction

Despite substantial efforts over several decades to promote the increased intake of fruits and vegetables (FV), only 12% of American adults meet the Dietary Guideline’s recommendations for fruit intake and 9.3% meet the recommendations for vegetable intake [1]. A low intake of FV puts adults at higher risk of multiple chronic diseases [2,3]. Disparities in diet quality by income and by Supplemental Nutrition Assistance Program (SNAP) participation persist, with higher-income individuals and income-eligible people not participating in SNAP reporting higher diet quality, including more mean daily cups of FV compared to SNAP participants (5.2 cups/day, 4.6 cups/day, and 3.7 cups/day, respectively) [4]. Prior to the COVID-19 pandemic, the United States had higher rates of food insecurity among households with children than nearly every other Organization for Economic Cooperation and Development nation [5], and multiple authors have reported increases in the rates of food insecurity since the COVID-19 pandemic began in 2020 [6,7,8]. The USDA Economic Research Service found a 1.3% increase in food insecurity among households with children in 2020 [9]. While studies have found SNAP to be helpful in reducing food insecurity among participating households, food insecurity and economic hardship persist among program participants [10,11,12]. In response to reports by the Institute of Medicine and multiple others that SNAP benefits are too low to allow participants to afford a healthy and secure diet in most places across the United States [13,14,15], the United States government updated the Thrifty Food Plan in 2021, which led to an increase of about 25% in SNAP benefit levels, the largest in the program’s history [16]. Additional resources notwithstanding, economically disadvantaged neighborhoods often have limited access to fresh produce [17]. Greater efforts are needed to offer SNAP participants opportunities to increase FV consumption while supporting local food systems. One such strategy is the provision of financial incentives for the purchase of locally grown FV at farmers’ markets and other retail outlets.

Congress established the Food Insecurity Nutrition Incentive (FINI) program in the Agricultural Act of 2014 (P.L. 113-79) [18], offering $86.1 million in grants to organizations to provide SNAP participants with these incentives for FV purchases. The 2018 Farm Bill continued funding for FINI, renaming it the Gus Schumacher Nutrition Incentive Program (GusNIP) and providing increased and permanent funding. While GusNIP programs vary substantially across the United States [19], most participating retailers have the following program characteristics: (1) offer incentives to participants every day the retailer is open; (2) use a $1:$1 match rate, and (3) impose a maximum on the incentive value that can be redeemed, the majority of which are set at $20 per day [20]. Program funding is limited, and retailer participation and subsequent shopper access to these incentive benefits are only available to a small fraction of people participating in food assistance programs.

The California Nutrition Incentive Program (CNIP), the state’s FINI program, is funded by a federal GusNIP grant matched with state funds and was established by Assembly Bill 1321 “for the purposes of encouraging the purchase and consumption of California fresh fruits, nuts, and vegetables by directly linking California fresh fruit, nut, and vegetable producers with nutrition benefit clients” [21]. CNIP grantees are primarily operators of farmers’ markets and are provided flexibility to structure programs to suit local conditions, especially considering the limited resources available for incentives. Local programs are responsible for program marketing, design, and implementation. In farmers’ markets with higher numbers of weekly SNAP redemptions, maximum point-of-purchase dollar-for-dollar incentive levels tend to be low, often $5–$10 per market day. In areas with fewer SNAP shoppers, some markets offer incentives of $20 or more per market day.

The Food Insecurity Nutrition Incentive programs were inspired by the successful Healthy Incentives Pilot (HIP) in Massachusetts. HIP found SNAP participants receiving a 30% rebate on purchases of FV in supermarkets and superstores increased their FV intake by about 0.24 cup-equivalents compared to non-participating SNAP participants [22]. The largest evaluation of subsequent point-of-purchase incentive programs in farmers’ markets and grocery stores nationally, conducted by Westat, found that FINI shoppers received on average $15–$23 in incentives during their last shopping trip and that the program had a positive impact on household FV expenditures, ranging from $9 to $15 monthly [20]. In a preliminary report, the same authors reported that FINI did not have an impact on FV consumption and that awareness of FINI programs is low, reporting that fewer than 40% of SNAP participants living near retailers offering match incentive programs knew about them [23]. Most other studies of farmers’ market incentive programs have used small sample sizes and many use self-reported measures and weak study designs. The most consistent finding related to the impact of point-of-purchase incentive programs on participants is related to produce expenditures and/or purchases, and studies consistently report these programs are associated with an increase in produce purchases among participants [20,24,25,26]. The impact of incentive programs on produce consumption and food security, however, is less clear. Some studies have found associations between incentive programs and quantitatively measured fruit and/or vegetable consumption [22,27,28], though others have not found consistent associations between incentive programs and measured produce consumption, despite some of these studies finding that participants self-report perceived increases in produce consumption due to the programs [29,30,31]. Few studies have examined the impact of incentive programs on food security [25,27,31]. One small study of 54 predominantly white women in Utah measured food insecurity using the USDA 6-item food security scale in a pretest/posttest design and found that fewer people reported experiencing food insecurity four weeks after their initial participation in a dollar-for-dollar incentive program matching up to $10 per farmers’ market visit [31]. Studies have identified the benefits of incentive programs beyond consumers, including the increased use of food assistance benefits and associated revenue at participating markets [32,33,34] and higher overall FV expenditures [20]. Simulation models have also estimated that the widespread expansion of FV incentive programs would contribute to improved health outcomes and significant societal cost savings [35,36]. Finally, while incentive levels vary across the country, few studies have explored the impact of the amount of incentive available to participants and outcomes related to produce purchases, consumption, and/or food security.

The present CNIP study was an early evaluation of a statewide program directed to an ethnically diverse California population. The evaluation sought to understand SNAP participants’ perceptions and awareness of CNIP and to assess differences in produce consumption, food security, and proportion of produce bought for households at farmers’ markets among SNAP participants shopping at farmers’ markets offering $10 or $20 per visit maximum dollar-for-dollar point of purchase incentives, compared to SNAP participants shopping at farmers’ markets and supermarkets which were not offering incentives (we use the term “supermarket” throughout to refer to both chain supermarkets as well as independent large grocery stores with full service produce departments). At the time of this evaluation, no supermarkets in California participated in the incentive program, and thus we were unable to compare the level of incentive offered at different types of retail outlets. Nevertheless, this is among the first studies exploring differences in outcomes among farmers’ market programs with different incentive benefit levels. Information from this early evaluation is intended to assist program planners and implementers in refining programs to achieve desired program outcomes.

## 2. Materials and Methods

### 2.1. Study Design

Our team collected the data during the summer of 2018 (June–August) using a cross-sectional study design. We selected a convenience sample of farmers’ markets, three with a $10 maximum point-of-purchase CNIP incentive, three with a $20 maximum CNIP incentive, and five comparison farmers’ markets (not offering incentives) for participation based primarily upon the market’s average number of weekly SNAP redemptions (to ensure data collectors would encounter enough SNAP shoppers eligible to participate in the study, we tried to visit the markets with the highest number of weekly SNAP redemptions in each group). We also considered the percent of SNAP-eligible people that spoke English and/or Spanish in the census tract in which the farmers’ markets were located, to be sure that our bilingual English/Spanish team would have the capacity to interview participants in the appropriate language, but no markets were excluded based upon language characteristics. Nine comparison supermarkets (not offering incentives) were selected based upon location near selected farmers’ markets (within a 15-mile radius) and census tract percentage of SNAP-eligible people. At each market, a convenience sample of up to 30 shoppers was recruited to complete interviewer-administered intercept surveys in English or Spanish.

### 2.2. Participants and Recruitment

At farmers’ markets accepting SNAP benefits, SNAP participants need to visit the market manager to swipe their Electronic Benefit Transfer (EBT) card in exchange for market tokens. Market managers referred SNAP participants requesting tokens to the data collection team which was stationed nearby. Supermarket participants were approached upon entering or exiting the store. Shoppers were informed about the study and invited to participate if they were adults, English- or Spanish-speaking, and members of a household receiving SNAP benefits. Shoppers recruited at intervention farmers’ markets were required to have used the incentive program at that market at least once within the past month. Shoppers recruited at farmers’ markets not participating in the incentive program were ineligible to participate if they had used an incentive at another farmers’ market within the past month. After completing the survey, all participants received a $10 gift card.

All study procedures were approved by exempt review by the Institutional Review Board at {blinded for peer review}. Oral consent was obtained from all participants in English or Spanish as appropriate.

### 2.3. Procedures

Interviewer-administered surveys, which took 15–20 min, were completed on-site at the farmers’ market or supermarket, near the market manager’s station at farmers’ markets, and at the store entrance/exit at supermarkets. While our data collectors conducted interviews in public, they tried to find as quiet and a private a space as possible for conducting the interviews. We offered small children coloring books and crayons to reduce distractions.

### 2.4. Instruments

The survey measured FV consumption among the shoppers intercepted using questions from the National Health and Nutrition Examination Survey Dietary Screener Questionnaire (NHANES DSQ) [37,38] and measured household food security using the United States Department of Agriculture 6-item household food security module [39]. In addition, the survey included questions about the number of times participants shopped at the farmers’ market in the past month, the dollar value of CNIP incentives they used the last time they shopped there, and the perceived importance of CNIP. Additional questions assessed participants’ produce purchasing behaviors and sociodemographic characteristics. We also asked supermarket shoppers about their familiarity with CNIP and their interest in shopping at farmers’ markets participating in CNIP after learning about the program. At each market, one interviewer conducting interviews in English and one interviewer conducting interviews in Spanish also asked participants a short series of open-ended questions regarding their perceptions of CNIP and the impact it had on their families (most markets had three to five total interviewers). These questions were asked after participants completed the quantitative section of the survey and captured their experience with and perceptions of CNIP, along with their opinions about the importance of California-grown produce. Data collectors used structured interview guides to create a more standardized approach to understanding themes reported among this group. Open-ended questions were audio-recorded and transcribed. Interviews conducted in Spanish were translated after transcription using an online service.

### 2.5. Measures

We calculated household food security using a standard protocol to code and sum responses to the 6-item scale. Scores of 2–6 points were categorized as food insecure and scores of 0–1 points were categorized as food secure [40]. We converted responses to the NHANES DSQ questions about produce consumption into quantitative estimates of cup equivalents of FV consumed per day using publicly available scoring algorithms [41]. The number of incentive dollars received in the past 30 days was estimated by multiplying the dollar value of CNIP incentives participants reported using the last time they used the program at a farmers’ market by the number of times they shopped at that market in the past 30 days.

### 2.6. Data Analysis

**Figure 1:**
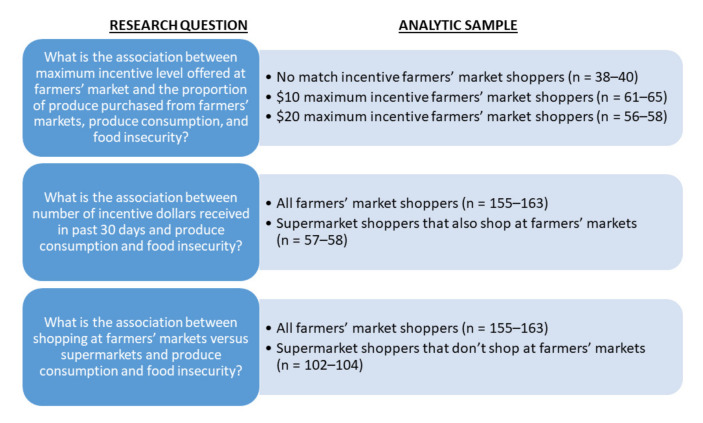
Analytic sample used to answer each research question in the California Nutrition Incentive Program evaluation study n’s vary due to different outcomes having slightly different analytic sample sizes due to variables required to run the models.

Figure 1 describes the analytic sample used to answer each of the research questions. Respondents were excluded from analysis if they had missing information on covariates, did not report identifying as male or female (due to sex-based scoring algorithms for the validated produce intake measure used), or were recruited at supermarkets and did not state whether they also shopped at farmers’ markets. Due to missing data, participants surveyed at supermarkets who reported also shopping at farmers’ markets were excluded from the analyses assessing produce consumption and food security outcomes by maximum incentive level offered; the maximum incentive offered at the markets where they shopped was not known.

We conducted a multilevel linear regression to investigate the associations between the exposures of interest (maximum incentive level offered at farmers’ markets and the number of incentive dollars received in the past 30 days) and continuous outcomes, including intakes of fruits, vegetables, and legumes (excluding fried potatoes). We also used multilevel logistic regression to examine the associations between exposures of interest and binary outcomes, including whether participants reported purchasing more than half of their produce at farmers’ markets, were food insecure, or exhibited elements of food insecurity (having food they bought not last, not being able to afford to buy balanced meals, cutting the size of or skipping meals, eating less than felt they should, or being hungry but not eating). Models investigating the associations between the maximum incentive level offered at farmers’ markets and outcomes of interest compared participants based solely on the maximum incentive offered at the farmers’ market they were intercepted at to understand the effect of setting different maximum incentive levels. In contrast, models examining the associations between the number of incentive dollars received in the past 30 days and outcomes of interest used the actual amount of incentive participants reported receiving to understand how the degree of program utilization affected outcomes. All models were adjusted for age, gender, race/ethnicity, education, income, employment, and household size, as well as for clustering by market using the Taylor linearization series method. Significance was set at α < 0.05 [42,43]. Data were analyzed using SAS v9.4 (Cary, NC, USA).

### 2.7. Qualitative Data Analysis

The methodologies used to guide our approach to analyzing the qualitative data are phenomenology and implementation science. Phenomenology allows us to analyze the data collected in our open-ended questions by aiming to create meaning from participants’ lived experiences and their perceptions of certain topics, such as CNIP and California-grown produce. Phenomenology tells us that reality is captured within the lived experiences participants report [44]. Additionally, our methods are also guided by implementation science, which allows us to use our findings to improve the quality and effectiveness of an intervention. The ultimate goal of our open-ended questions was to use participant feedback to inform the execution of CNIP [45].

To analyze the responses to our open-ended questions, we used a process of descriptive coding to find emerging themes within our data. For open-ended responses, two authors reviewed all transcripts, both of whom had previous experience and training coding qualitative interviews. Together, they created a structured codebook that identified the main themes from the responses. One author applied the descriptive codes to sentences and short phrases from the data and used an Excel spreadsheet to tally the frequency of each code. The other author reviewed the coding and then the two discussed discrepancies until an agreement was reached. Both authors then read through all coded responses to identify six main themes within our data and performed a content analysis using the frequency of the codes to determine general trends in the qualitative data [44,45].

## 3. Results

Surveys were completed by 163 SNAP participants intercepted at farmers’ markets and 162 SNAP participants intercepted at supermarkets not participating in CNIP (Table 1). A total of 16% of the shoppers approached declined to participate in the study; of those who agreed to participate, 11% did not meet inclusion criteria. We invited 54 of the survey respondents intercepted at farmers’ markets participating in CNIP to complete an additional open-ended qualitative interview and all but 4 agreed.

Participants were, on average, 40 years of age and belonged to a household consisting of 3 to 4 people (Table 1). The distribution of participants’ gender, race/ethnicity, and education status varied by type of market. A higher proportion of participants intercepted at farmers’ markets were female and more highly educated than those intercepted at supermarkets. Over one-third of the participants recruited at supermarkets reported also shopping at farmers’ markets.

### 3.1. Use, Perception, and Awareness of the Incentive Program

Among participants intercepted at farmers’ markets participating in CNIP, most (79%) reported that the incentive program was very important to their decision to shop at farmers’ markets (Table 1). Almost all (99%) participants shopping at farmers’ markets offering a $10 maximum incentive and about two-thirds (66%) of those at markets offering a $20 maximum incentive received the maximum incentive amount offered the last time they shopped. Most participants interviewed at supermarkets (82%) were not aware of CNIP; however, when told about the program, nearly all said that they were very likely (59%) or somewhat likely (37%) to shop at the farmers’ market after learning about the incentive program (Table S1).

### 3.2. Produce Purchasing Behavior

The incentive benefit level available at the farmers’ market was significantly associated with study participants reporting they purchased more than half of their produce from farmers’ markets (Table 2). Participants shopping at markets offering $20 maximum incentives had more than three times the odds (3.144 (95% CI: 1.138, 8.681) of reporting that they purchase more than half their produce at farmers’ markets compared to participants shopping at markets with $10 maximum incentives, and over eight times the odds (8.113 (95% CI: 2.218, 29.675) of reporting they purchase more than half of their produce at farmers’ markets compared to participants shopping at farmers’ markets not offering any incentive.

### 3.3. Produce Consumption

Notably, shoppers at farmers’ markets with or without incentive programs reported consuming significantly more FV (0.548 cup equivalents per day 95% CI: 0.137, 0.960) than participants who did not shop at farmers’ markets (Table 2). No significant differences in produce consumption were found among shoppers at farmers’ markets regardless of whether the market offered no incentive, a $10 maximum incentive, or a $20 maximum incentive, indicating no program effects on FV consumption by shoppers. The analysis also showed that the number of incentive dollars received by participants in the past 30 days was not associated with any significant differences in their FV consumption (Table 3).

### 3.4. Food Security

We did not find a significant difference in the odds of experiencing household food insecurity between participants shopping at farmers’ markets and those not shopping at farmers’ markets; however, farmers’ market shoppers did have lower odds of exhibiting multiple indicators of food insecurity (Table 2), regardless of whether they shopped at farmers’ markets offering incentives.

There was no significant difference in overall food security between participants shopping at farmers’ markets offering different maximum incentive levels (none, $10, or $20), although one indicator of food security was significantly different: participants shopping at markets offering $10 maximum incentives had 0.437 (95% CI: 0.236, 0.811) times the odds of being hungry and not eating compared to participants shopping at markets not offering any incentive (Table 2).

In our analysis exploring the relationship between the number of incentive dollars participants received in the past 30 days and food security, we found significant differences (Table 3). Each additional incentive dollar received by farmers’ market shoppers was significantly associated with lower odds of being food insecure (OR (95%CI): 0.987 (0.976, 0.999)). This difference appeared driven by two components of food security: lower odds of shoppers reporting that they ate less than they felt they should (OR (95%CI): 0.988 (0.978, 0.998)) and that they were hungry but did not eat (OR (95%CI): 0.982 (0.968, 0.996)).

### 3.5. Qualitative Findings

Content analysis of open-ended responses to questions about participants’ experiences with CNIP identified six themes (Table 4). Overall, participants expressed consistently positive experiences with the program. Most participants reported that CNIP enabled them to increase the quantity of FV they were able to buy and that the program allowed them to purchase higher quality and fresher FV. Participants reported that CNIP helped them to support their local communities and to support sustainable food production practices. Many participants felt that CNIP encouraged them to buy a wider variety of produce items and enabled them to try new FV. Every participant in the sample expressed appreciation for the program and a desire to see it expand.

## 4. Discussion

The Supplemental Nutrition Assistance Program benefit levels have long been known to be inadequate, particularly in parts of the country with a high cost of living, leading to disincentives to purchase nutrient-dense but calorie-limited healthy foods such as FV [13,14]. The Biden administration’s recent change to the funding formula that has increased benefit levels likely will help alleviate some of these challenges. Still, SNAP incentive programs provide participants with modest amounts of additional money, aiming specifically to increase FV purchases and consumption. Although the program aims to increase produce consumption among SNAP household members, it is also possible for participants to use the benefits to purchase their usual produce items, freeing up additional funds for other food items. This utilization of the program may not increase produce consumption but could improve food security, which could explain the result observed in our study. We found that SNAP participants shopping at farmers’ markets with access to higher incentives had significantly better food security, though no significant increase in produce consumption. It is important for new studies implemented after the SNAP benefit increase to explore whether the additional benefits make it easier for participants to meet their food security needs while also being able to purchase greater quantities of FV. However, recent food price increases due to supply chain limitations and other inflationary triggers may erode some of the real value of recent SNAP benefit increases, as has been seen in other recent periods [46].

Prior studies have found these incentive programs to be associated with increased FV purchases and spending [47,48]. Our study found that SNAP participants shopping at farmers’ markets offering $20 maximum incentives were more likely to report purchasing more than half of their produce at farmers’ markets than those shopping at markets with $10 incentives, while shoppers at markets not providing incentives were the least likely to report purchasing more than half their produce at farmers’ markets. While differences in the demographic characteristics (both unmeasured and measured) of participants shopping at the markets offering different incentive levels may partially explain these results, the dose-response relationship observed after adjusting for measured characteristics suggests the finding may be robust, especially given the (measured) demographic similarities among participants interviewed at markets not providing incentives and those providing $20 incentives. We also found that nearly all participants shopping at markets offering a $10 maximum incentive reported using the entire amount of the incentive when they shopped and 66% of participants at markets offering a $20 maximum used the entire amount. Since many participants redeem the full amount, higher incentives may help programs to have a greater positive impact on local producers and food systems, if incentive levels offered contribute to increased SNAP purchases at farmers’ markets. Again, it is important to consider whether demographic differences in the populations partially explain these findings despite our models adjusting for those differences and acknowledge the possibility that some SNAP participants may not need the full $20 incentive to fulfill their FV needs. However, it is also possible that these results reflect that some participants may not be able to afford to spend $20 worth of their SNAP benefit on produce on any given day. Higher incentive match levels such as a 2:1 match instead of a 1:1 match may also yield a greater positive impact. Other studies have found that incentive programs positively impact farmers’ market sales and farmers [49,50,51]. The next step for research analyzing incentive programs is to explore the relationship between incentive levels offered, SNAP participants’ monthly benefit levels, and how the interaction of these factors contributes to program impacts on SNAP shoppers and farmers’ markets and vendors/growers.

While incentive programs have been associated with increases in FV consumption in a number of studies, beginning with the Healthy Incentives Pilot [52] that led to the FINI and GusNIP programs, the literature is somewhat mixed on these programs’ impact on produce intake [29,30]. Our study found in qualitative interviews that participants perceived the CNIP program to have increased their consumption of FV. While our quantitative measures of produce consumption found that farmers’ market SNAP shoppers reported consuming more produce than SNAP shoppers not using farmers’ markets, we did not find any differences in produce consumption among SNAP participants shopping at farmers’ markets offering no incentive, a $10, or $20 maximum incentive. These findings are consistent with those reported by Olsho et al. in a study of a farmers’ market incentive program in New York City in 2011 [29]. That study also found that farmers’ market shoppers reported consuming more servings of FV than those not shopping at farmers’ markets, but no consumption pattern related to whether people knew about or used the incentive program, and that participants were more likely than non-participants to self-report a retrospective increase in FV consumption. A study of the Philly Food Bucks program also found that participants were more likely than non-participants to self-report that they had increased their FV consumption [51]. Given that prior studies have found relatively small household increases in produce spending and/or the number of servings of FV purchased monthly as a result of incentive programs [48], it is plausible that program participants are buying and eating more produce, but not enough more to be detected by the methods being used to assess produce consumption in our study and many others [53]. Future studies should consider using methods more sensitive to picking up smaller differences in intake, such as 24-h recalls. Because the match incentive is spread across family members and most participants in our study had multiple people in their households, changes in FV consumption may not have been large enough for the individual study participant to be captured in our results. It is possible that higher levels of the newly purchased produce items were distributed to other household members, such as children or elders with health issues, but that was beyond the scope of the current study. The qualitative perceptions reported may be due to participants feeling that eating even one more serving of produce per week as a result of the program is significant, even if it is not detectable with our quantitative study methods.

While our study did not find a strong relationship between the maximum match incentive level available at the farmers’ market and food security status, we did find a relationship between food security and participants’ reported use of incentive program benefits. For each additional dollar of incentive participants received, there was an associated 1.3% decrease in the odds of being food insecure. A few other studies have assessed the impact of incentive programs on food security. The Westat evaluation of the national FINI program did not report any program impact on adult food security in a preliminary report [23]; however, a couple of smaller studies have reported a positive relationship between incentive program use and food security [27,31]. The relationship observed in our study could be related to higher levels of available incentives being more beneficial to participants’ food security outcomes. However, this finding also could be due to unobserved differences in participants who are able to spend more of their SNAP benefits on produce at farmers’ markets. This could be related to the dollar value of SNAP benefits participants receive on a monthly basis and/or the way in which participants are able to budget their SNAP dollars. We did not ask participants the monthly value of SNAP benefits they receive, but future studies assessing the relationship between incentive programs and food security may consider assessing whether these programs are more beneficial to participants who receive larger monthly SNAP benefits. Because increasing food security is an important goal for the SNAP program and incentive programs, it is essential to understand how to optimally design programs to improve food security.

Our qualitative findings are similar to those of other studies that find incentive program participants like and appreciate the program [23,24]. Our study respondents consistently appreciated participating in CNIP. We heard positive feedback about the program, how it helps participants buy more FV, try new FV, and feel good about supporting local farmers and communities. Given our low study refusal rate (16%) and high agreement among those invited to complete the qualitative interviews (93%), this positive experience may well reflect how most SNAP participants who shop at farmers’ markets and utilize these incentive programs feel about them, though selection bias, as well as social desirability bias, may have inflated the positive reporting here. An important aspect for future studies to consider studying is the impact of this program on SNAP participants’ well-being. As has been seen in other studies [54], participants in our study who were not shopping at farmers’ markets offering the incentive program had little awareness of the benefit [55].

### 4.1. Limitations

Multiple factors limit our study. We used a cross-sectional study design which prevents our ability to draw causal inferences. Additionally, study participants were recruited from a convenience sample of markets participating and not participating in CNIP, which limits the generalizability of our findings. Due to the statewide implementation of the program, there were demographic differences among participants using farmers’ markets with different levels of incentives or no incentives. While our models adjusted for some of these differences, it is possible that unmeasured characteristics may confound our analysis, such as attitudes and preferences towards FV and related behaviors (e.g., liking of FV and valuing and/or enjoying cooking). While we used validated measures of produce consumption and food security, short screening measures may have lacked sensitivity to detect small differences in these outcomes. Our open-ended interviews revealed strong positive experiences with CNIP and multiple reports of benefits to participants’ health, food security, and ability to positively impact their communities, yet these data are subject to social desirability bias. The strengths of our study include our exploration of the association of different maximum incentive levels with produce consumption and food security outcomes using validated measures. Additionally, the mixed methods we used to assess both quantitative and qualitative experiences of SNAP participants with and without access to CNIP are also a strength.

### 4.2. Implications for Research and Practice

Increasing funds to help SNAP participants gain access to and afford an adequate and healthy diet has been proposed as an important way to improve health and nutrition and reduce disparities, and was adopted by the federal government in August 2021. Incentive programs such as CNIP have become popular among advocates and policy makers and are greatly appreciated by program users, so may continue to play a role in increasing access and incentives to purchase FV. Consistent evidence suggests these programs increase purchases of local fresh produce and expand the use of farmers’ markets among SNAP beneficiaries, which benefits state agriculture and local food systems. While program participants self-report that participation increases their produce purchasing and consumption, differences in respondents’ self-reported dietary intake were not detected quantitatively in our study and have been inconsistent across studies. More resource-intensive evaluations may be required to measure and assess the relationship between purchasing and consumption, and to measure consumption among all household members (not limited to shoppers) using more detailed dietary intake measures to capture program impacts on households. Our findings suggest that more effective marketing of the incentive program is needed to inform and reach eligible SNAP participants to utilize the program and to promote the use of all of the incentive dollars they receive for produce. Better understanding of how to design and fund incentive programs for maximum impact on produce consumption is important.

The United States is currently experiencing high levels of food insecurity and high rates of diet-related chronic diseases and needs strong interventions to reduce them. Despite not finding a clear relationship between the maximum incentive level offered and food security, our study suggests there may be a relationship between the degree of incentive program use and food security. Additionally, our findings suggest that having the incentive program available at farmers’ markets may encourage SNAP shoppers to purchase more of their produce at farmers’ markets, strengthening local food systems and increasing the chances participants are purchasing organic produce, which tends to be more accessible and affordable in farmers’ markets [56]. Future large, rigorous studies are needed to assess whether the current investments in incentive programs adequately benefit participants’ nutrition and food security and whether additional interventions, including higher incentives, may be needed to improve outcomes for all stakeholders. Further, SNAP participants underutilize incentive programs. Additional work needs to be considered to make benefits more equally available to all, especially given the high reported value among program participants.

## Figures and Tables

**Table 1:** Demographic characteristics of participants in the California Nutrition Incentive Program evaluation study by market type ^1^.

Demographic Characteristic	Farmers’ Market Shoppers				Supermarket Shoppers		All Shoppers (*n* = 325)
	No Match Incentive (*n* = 40)	$10 Maximum Incentive (*n* = 65)	$20 Maximum Incentive (*n* = 58)	Total Farmers’ Market Shoppers (*n* = 163)	Supermarket Shoppers That Do Not Shop at Farmers’ Markets (*n* = 104)	Supermarket Shoppers That Also Shop at Farmers’ Markets (*n* = 58)	
Age (Mean (SE))							
Age	38.7 (2.3)	39.0 (2.6)	41.4 (3.8)	39.8 (1.6)	40.3 (1.4)	39.7 (0.7)	39.9 (0.9)
Gender (% (SE))							
Female	72.5% (5.0)	89.2% (7.4)	79.3% (2.0)	81.6% (3.9)	61.5% (6.2)	77.6% (7.5)	74.5% (3.9)
Race/ethnicity (% (SE)) ^2^							
Hispanic	32.5% (8.4)	70.8% (27.4)	13.8% (2.3)	41.1% (13.7)	45.2% (13.0)	46.6% (12.6)	43.4% (9.0)
Non-Hispanic White	50.0% (10.4)	21.5% (22.0)	60.3% (4.9)	42.3% (10.6)	36.5% (9.7)	34.5% (10.5)	39.1% (6.8)
Non-Hispanic Black/African American ^3^	5.0% (5.8)	1.5% (1.6)	8.6% (3.1)	4.9% (2.1)	2.9% (1.2)	1.7% (1.8)	3.7% (1.2)
Non-Hispanic Other	12.5% (8.3)	6.2% (4.0)	17.2% (5.1%)	11.7% (3.3)	15.4% (4.3)	17.2% (3.9)	13.8% (2.6)
Education (% (SE)) ^2^							
High school graduate, GED, or less	35.0% (6.9)	55.4% (22.7)	13.8% (3.8)	35.6% (10.9)	63.5% (3.4)	51.7% (12.2)	47.4% (7.0)
Associate’s degree, vocational certificate, or some college	30.0% (8.7)	24.6% (7.0)	48.3% (5.7)	34.4% (5.1)	28.8% (2.7)	32.8% (8.0)	32.3% (3.2)
Bachelor’s degree or higher	35.0% (11.8)	20.0% (15.8)	37.9% (9.3)	30.1% (7.6)	7.7% (2.3)	15.5% (8.2)	20.3% (4.9)
Income (% (SE)) ^2^							
Less than $10,000	42.5% (11.6)	47.7% (11.1)	29.3% (1.3)	39.9% (5.8)	46.2% (5.9)	36.2% (5.5)	41.2% (3.3)
$10,000–$19,999	40.0% (6.1)	33.8% (4.9)	41.4% (6.5)	38.0% (3.2)	30.8% (5.0)	31.0% (6.5)	34.5% (2.6)
$20,000 or more	17.5% (8.6)	18.5% (10.9)	29.3% (6.1)	22.1% (5.1)	23.1% (4.4)	32.8% (8.1)	24.3% (2.8)
Employment Status (% (SE)) ^2^							
Employed full-time	15.0% (7.0)	9.2% (6.0)	15.5% (3.7)	12.9% (3.1)	12.5% (4.7)	12.1% (6.3)	12.6% (2.6)
Employed part-time	22.5% (15.2)	23.1% (14.4)	25.9% (3.2)	23.9% (6.1)	24.0% (5.5)	25.9% (5.1)	24.3% (3.6)
Unemployed seeking employment	27.5% (9.9)	13.8% (1.5)	17.2% (2.8)	18.4% (3.5)	27.9% (7.2)	20.7% (8.9)	21.8% (3.4)
Not employed and not seeking employment	35.0% (8.9)	53.8% (20.6)	41.4% (4.0)	44.8% (8.2)	35.6% (6.5)	41.4% (8.0)	41.2% (4.6)
Household Size (Mean (SE))							
Household Size	2.5 (0.6)	3.8 (0.9)	2.8 (0.3)	3.1 (0.4)	3.7 (0.3)	3.8 (0.5)	3.4 (0.3)
Incentive program use, perceived value, and reported produce purchasing behavior (% (SE))							
Reported that Market Match was “Very Important” to their decision to shop at the farmers’ market	N/A ^4^	78.5% (8.3)	79.3% (2.0)	N/A ^4^	N/A ^4^	N/A ^4^	N/A ^4^
Received maximum Market Match incentive at farmers’ market	N/A ^4^	98.5% (1.4)	65.5% (5.7)	N/A ^4^	N/A ^4^	N/A ^4^	N/A ^4^
Reported mechanism for learning about the incentive program (% (SE))							
Information at farmers’ market during a prior visit	N/A ^4^	49.2% (11.3)	70.7% (6.1)	N/A ^4^	N/A ^4^	N/A ^4^	N/A ^4^
Friend or family member	N/A ^4^	24.6% (1.4)	15.5% (3.7)	N/A ^4^	N/A ^4^	N/A ^4^	N/A ^4^
County social services or other agency	N/A ^4^	16.9% (4.9)	8.6% (0.1)	N/A ^4^	N/A ^4^	N/A ^4^	N/A ^4^
Other ^3^	N/A ^4^	21.5% (3.3)	8.6% (6.0)	N/A ^4^	N/A ^4^	N/A ^4^	N/A ^4^

^1^ Descriptive statistics adjusted for clustering by market, ^2^ Percents may not add up to 100% due to rounding, ^3^ Due to small cell sizes, the Non-Hispanic Black/African American category was combined into the Non-Hispanic Other category in the analytic models. ^4^ N/A indicates not applicable.

**Table 2:** Comparisons of reported produce purchasing behavior, produce consumption, and food insecurity by market type: farmers’ market and supermarket shoppers participating in the California Nutrition Incentive Program evaluation study.

	$10 Maximum Incentive Farmers’ Market Shoppers vs. No Match Incentive Farmers’ Market Shoppers		$20 Maximum Incentive Farmers’ Market Shoppers vs. No Match Incentive Farmers’ Market Shoppers		$20 Maximum Incentive Farmers’ Market Shoppers vs. $10 Maximum Incentive Farmers’ Market Shoppers		All farmers’ Market Shoppers vs. Supermarket Shoppers That Do Not Shop at Farmers’ Markets	
Reported produce purchasing behavior	*n*	Odds Ratio ^1^	*n*	Odds Ratio ^1^	*n*	Odds Ratio ^1^ (95% Confidence Interval)	*n*	Odds Ratio ^1^ (95% Confidence Interval)
		(95% Confidence Interval)		(95% Confidence Interval)				
Reported purchasing more than half their produce at farmers’ market	105	2.581	98	**8.113**	123	**3.144**	N/A ^2^	N/A ^2^
		(0.610, 10.922)		**(2.218, 29.675)**		**(1.138, 8.681)**		
Produce consumption (cup equivalent per day)	*n*	Beta Coefficient ^3^	*n*	Beta Coefficient ^3^	*n*	Beta Coefficient ^3^	*n*	Beta Coefficient ^3^
		(95% Confidence Interval)		(95% Confidence Interval)		(95% Confidence Interval)		(95% Confidence Interval)
Fruit	105	−0.077	98	0.088	123	0.165	267	0.155
		(−0.274, 0.121)		(−0.268, 0.444)		(−0.156, 0.485)		(−0.101, 0.411)
Vegetables and legumes (NOT fried potatoes)	105	0.122	98	−0.088	123	−0.210	267	**0.312**
		(−0.082, 0.325)		(−0.284, 0.108)		(−0.466, 0.046)		**(0.098, 0.526)**
Fruit, vegetables, and legumes (NOT fried potatoes)	105	0.06	98	−0.005	123	−0.065	267	**0.548**
		(−0.302, 0.421)		(−0.523, 0.513)		(−0.618, 0.488)		**(0.137, 0.960)**
Food insecurity	*n*	Odds Ratio ^1^	*n*	Odds Ratio ^1^	*n*	Odds Ratio ^1^	*n*	Odds Ratio ^1^
		(95% Confidence Interval)		(95% Confidence Interval)		(95% Confidence Interval)		(95% Confidence Interval)
Food insecure	99	0.757	94	0.76	117	1.004	257	0.575
		(0.448, 1.280)		(0.387, 1.493)		(0.436, 2.308)		(0.308, 1.074)
Food bought did not last	104	1.05	98	1.46	122	1.391	265	**0.441**
		(0.422, 2.615)		(0.558, 3.821)		(0.657, 2.942)		**(0.215, 0.904)**
Could not afford to	102	1.465	96	1.15	122	0.785	264	0.623
buy balanced meals		(0.855, 2.510)		(0.440, 3.010)		(0.309, 1.997)		(0.284, 1.369)
Cut the size of or skipped meals	105	0.814	97	0.586	122	0.721	266	**0.525**
		(0.489, 1.354)		(0.275, 1.249)		(0.269, 1.931)		**(0.304, 0.906)**
Ate less than felt should	104	0.846	98	0.626	122	0.74	266	0.621
		(0.536, 1.333)		(0.298, 1.315)		(0.326, 1.679)		(0.374, 1.033)
Were hungry but did not eat	103	**0.437**	97	0.478	120	1.092	263	**0.345**
		**(0.236, 0.811)**		(0.198, 1.151)		(0.416, 2.864)		**(0.159, 0.748)**

^1^ Odds ratios adjusted for age, gender, race/ethnicity, education, income, employment, household size, and clustering by market. Bold font indicates results statistically significant at *p* < 0.05. ^2^ N/A indicates not applicable. ^3^ Beta coefficients adjusted for age, gender, race/ethnicity, education, income, employment, household size, and clustering by market. Bold font indicates results statistically significant at *p* < 0.05.

**Table 3:** Adjusted ^1^ beta coefficients and odds ratios estimated from regression models assessing the relationship between the estimated number of incentive dollars received in the past 30 days and daily produce consumption (in cup equivalents per day from NCI fruit and vegetable screener) and food security (from USDA 6-item screener) in the California Nutrition Incentive Program evaluation study.

Produce Consumption (Cup Equivalents/Day)	(95% Confidence Interval)
Fruit (*n* = 221)	0.000 (−0.003, 0.004)
Vegetables and legumes (NOT fried potatoes) (*n* = 221)	0.000 (−0.002, 0.003)
Fruit, vegetables, and legumes (NOT fried potatoes) (*n* = 221)	0.001 (−0.005, 0.007)
Food Security	OR (95% Confidence Interval)
Cut the size of or skipped meals (*n* = 220)	0.990 (0.979, 1.000)
Ate less than felt should (*n* = 220)	**0.988 (0.978, 0.998)**
Were hungry but did not eat (*n* = 218)	**0.982 (0.968, 0.996)**
Food bought did not last (*n* = 220)	0.999 (0.988, 1.011)
Could not afford to eat balanced meals (*n* = 217)	0.996 (0.988, 1.005)
Food insecure (*n* = 212)	**0.987 (0.976, 0.999)**

^1^ Models adjust for age, gender, race/ethnicity, education, income, employment, and household size and clustering by market. Bold font indicates results statistically significant at *p* < 0.05.

**Table 4:** Themes, frequencies, and sample quotes to illustrate themes that emerged from qualitative analysis of a subsample of California Nutrition Incentive Program (CNIP) participants completing an open-ended survey module during the CNIP evaluation study (*n* = 50).

Theme	Supporting Quotations
Participants credit CNIP with helping them to eat more healthfully and improving their health	I’m eating better because I can afford to get fresh food, fresh vegetables and fruit that I wouldn’t get otherwise.When I was shopping in supermarkets, I wasn’t buying specifically fruits and vegetables, but here, the farmer’s market allows me to buy those fruits and vegetables and make food at home as opposed to buying more unhealthy foods that are processed and prepackaged.…we eat more fruits and vegetables this way. A lot more. (CNIP) has helped us to … eat a lot more fruits and vegetables instead of junk food.I think that for the household to be healthy, you have to be less stressed financially and this reduces the financial stresses for many families. Now, they can eat healthy, and it probably also affects their relationships with their families, friends, and communities.
CNIP has helped participants to be able to buy more food overall, as well as a greater quantity of fruits and vegetables	We’re able to get more food than money that we have because they match it, so we’re able to actually eat more fruits and vegetables and have enough food.We eat more fruits and vegetables because… we get more money to spend on produce instead of not having any food money budget left at the end of the month. I’m able to buy more of the kinds of foods that I like that I normally couldn’t afford or buy as many of them. So, it’s definitely been an amazing thing to happen and I’m so happy I found out about it and that is the reason I come (to the farmers’ market).”It’s really helped bring in larger amounts produce for less money, which is important when I’m budgeting with the EBT.I eat more fruits and vegetables because of this farmer’s market… it enables me to eat more of what’s good for me… It gives me a little bit more wiggle room to buy more of the fresh vegetables and things because it matches my dollar for dollar… It makes (the way my family eats) better because I can get more fruits and vegetables into my diet.
CNIP allowed participants to buy a wider variety of produce and enabled them to try new things	We get a little more variety. We’re more willing to try fruits and vegetables that we might not have otherwise because we’re getting the match. That’s been a good way to experiment with new flavors and trying to cook new things.We’re having so much variety with the fruits and vegetables that we’re able to get here… I’ll buy items even if we’re not used to eating it and we’ll experiment. And so, we’ll find new things that we can enjoy (that) I wouldn’t have if I hadn’t come here and seen such a great price on it.I’m able to have more variety of different choices of fruits and vegetables. I’m able to expand on different sorts of meals, not just be limited to which fruits and vegetables I can choose. As the seasons change, I’m able to buy different fruits and vegetables and try out different meals.
CNIP facilitated purchasing higher quality fruits and vegetables that were fresher	It offers us the ability to purchase higher quality food. It’s improved the types of food that we eat.It allows me to get organic and good produce, especially for my son. He always gets fresh food and I don’t think we would be able to do it without the match program.It allows me to feed my child more fresh foods every single day and without the… program, I don’t think I’d be able to do that.
Participants appreciate the opportunity CNIP provides them to engage with and support their local community	I think it’s been great. It’s encouraged me to bring my son out to the market and get involved in the community.It’s really important to support the local economy and support the local farmers as much as possible. Having the program really helps do that…I think we can all vote with our dollar and while these aren’t technically my dollars, that makes me feel more responsible because I’m receiving assistance and so I want to use those dollars as wisely as I can and put that money–cycle it back in.It’s just important to eat locally. You support your community, and they support you and that’s a good thing.
Participants expressed appreciation for CNIP and wanted to see the program expand	This is a very important program. It encourages people to spend money locally and support local growers in addition to allowing people to have better access to fruits and vegetables that are grown fresh. The match will allow people who have very little income or no income at all to be able to extend their spending dollars more.It’s been very important for my family. I have appreciated it and so I would definitely recommend continuing it. I think it’s good to promote to other families. I know that there’s probably a lot of families out there who don’t know about it yet and it could help them and so, I would recommend it for that too.I would say absolutely expand it because farmer’s markets are extremely important for both the consumers and the people selling the food because it helps put money back into the local farmers. People really need to start eating differently and I think that farmer’s markets encourage that because they have a lot of healthy food options. It’s definitely something that needs to be expanded. I think it should be everywhere.I think it’s an excellent program that I hope is available to more and more people in the future.Some of us really depend on (CNIP) to get fresh food for us and our kids and we feel so blessed that we’re able to do it.

## Data Availability

Data will be available from the corresponding author on reasonable request.

## Supplementary Materials

The following are available online at https://www.mdpi.com/article/10.3390/nu14132699/s1, Table S1: Awareness of and likelihood of using the California Nutrition Incentive Program among sampled supermarket shoppers (*n* = 162).
