# Supplementary material for: The California Nutrition Incentive Program: Participants’ Perceptions and Associations with Produce Purchases, Consumption, and Food Security
Source: Nutrients. 2022 Jun 29;14(13):2699. doi: 10.3390/nu14132699 (PMC9268255; doi:10.3390/nu14132699)
Supplement: Supplementary file 1 [file nutrients-14-02699-s001.zip › nutrients-1786453-supplementary.pdf]

Table S1. Awareness of and likelihood of using the California Nutrition Incentive Program among sampled supermarket shoppers (n = 162)

	% (SE)
Awareness of incentive program	
Had previously heard about it	17.9% (3.0)
Had not previously heard about it	82.1% (3.0)
Likelihood of using incentive program once heard about it	
Not at all likely	0.8% (0.8)
Not very likely	3.0% (1.5)
Somewhat likely	37.1% (4.2)
Very likely	59.1% (4.3)
